# Hysteroscopic Tubal Flushing Combined With Laparoscopic Milking for Ectopic Pregnancy Removal: A Case Report and Literature Review

**DOI:** 10.1002/ccr3.70812

**Published:** 2025-08-19

**Authors:** Azadeh Tarafdari, Marjan Ghaemi, Najmeh Nasiri Khormoji, Amirali Barkhordarioon, Shiva Hadizadeh, Mohammadamin Parsaei

**Affiliations:** ^1^ Department of Obstetrics and Gynecology, Imam Khomeini Hospital Complex Tehran University of Medical Sciences Tehran Iran; ^2^ Vali‐e‐Asr Reproductive Health Research Center, Family Health Research Institute Tehran University of Medical Sciences Tehran Iran; ^3^ School of Medicine Tehran University of Medical Sciences Tehran Iran; ^4^ Breastfeeding Research Center, Family Health Research Institute Tehran University of Medical Sciences Tehran Iran

**Keywords:** case report, ectopic pregnancy, hydrostatic pressure, hysteroscopy, laparoscopy, tubal pregnancy

## Abstract

We present a novel approach combining hysteroscopic flushing and laparoscopic milking for tubal ectopic pregnancy (EP) removal while preserving fallopian tubes. Postoperative beta‐human chorionic gonadotropin (β‐hCG) suspected remnant EP, necessitating methotrexate. Three‐month follow‐up confirmed bilateral tubal patency. This technique offers a tube‐preserving treatment for EP; however, β‐hCG monitoring is crucial.

## Graphical abstract

We report a 30‐year‐old woman with six weeks of amenorrhea and a suspected left tubal ectopic pregnancy (EP). Initially managed expectantly, she developed acute abdominal pain and rising beta‐human chorionic gonadotropin (β‐hCG), necessitating surgical intervention. A novel hysteroscopic tubal flushing technique was employed, utilizing saline infusion to dislodge the EP mass, followed by laparoscopic tubal milking. Postoperatively, the patient’s β‐hCG level decreased but later rose to 1,094 IU/L, suggesting possible remnant EP, prompting the administration of a single intramuscular dose of methotrexate. Three‐month follow‐up hysterosalpingography examination showed bilateral tubal patency with appropriate caliber and peristalsis. This suggested that laparoscopy‐assisted removal of tubal EP with hysteroscopic flushing appears to be a promising alternative to salpingectomy/salpingostomy for patients desiring to preserve their fallopian tubes intact. Nonetheless, vigilant β‐hCG monitoring is essential to detect any remnant pregnancies.

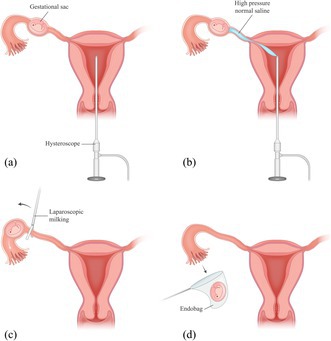

## 1 Introduction

Ectopic pregnancy (EP) occurs when the embryo implants and grows outside the uterine endometrial cavity, with a prevalence of 1%–2% in all pregnancies [1]. The fallopian tubes are the most common site for EPs, accounting for over 90% of cases [2]. This condition presents significant risks to maternal health, contributing to morbidity and mortality [3]. While advancements in the diagnosis and management of EPs have reduced the overall mortality rate, deaths from ruptured EPs remain a significant concern, accounting for up to 6.0% of maternal deaths [4].

Tubal EPs are primarily managed through two approaches: medical and surgical intervention. The primary medical approach involves methotrexate administration, a folate antagonist that inhibits nucleic acid synthesis in rapidly proliferating tissues, including developing embryonic cells [5]. Methotrexate serves as first‐line therapy for hemodynamically stable patients with confirmed or highly suspected EP when specific criteria are met: an unruptured mass, absence of fetal cardiac activity, and no absolute contraindications such as hemodynamic instability, coexisting intrauterine pregnancy, or breastfeeding [6]. A recent meta‐analysis reported a pooled treatment success rate of 79.3% for methotrexate therapy [7], with efficacy significantly influenced by factors such as the specific methotrexate regimen, initial beta‐human chorionic gonadotropin (β‐hCG) levels, EP mass size, and endometrial thickness [8, 9].

Surgical intervention is indicated when absolute contraindications to methotrexate exist or in cases of significantly elevated tubal rupture risks, such as fetal cardiac activity, high β‐hCG levels, or a gestational sac > 4 cm [6]. The surgical management of unruptured tubal EP typically involves two laparoscopic techniques of salpingectomy and salpingostomy. Salpingectomy removes the entire fallopian tube, which is often undesirable for patients seeking to preserve fertility [10]. In contrast, the more conservative salpingostomy involves incising the fallopian tube to extract the pregnancy tissue [10]. Although this method spares the fallopian tube and may be preferred by patients with future fertility plans, it carries a higher risk of incomplete tissue removal and recurrent EP [10, 11]. These limitations prompted us to develop a novel laparoscopy‐assisted technique using hydrostatic pressure via a hysteroscope to preserve the fallopian tube. We present a case of tubal EP managed with this fertility‐preserving method, following CARE guidelines [12].

## 2 Case History/Examination

A 30‐year‐old woman, gravida 2, para 0, with one prior abortion (G_2_P_0_Ab_1_), was referred to the Infertility Clinic at Vali‐e‐Asr Hospital, Imam Khomeini Hospital Complex, Tehran, Iran, with a suspected EP for further evaluation and management. After providing an explicit explanation of the purpose of our study, informed verbal and written consent was obtained from the patient for the anonymous publication of her personal medical information in this article.

At the time of the presentation to our center, the patient had experienced six weeks of amenorrhea since her last menstrual period. She reported regular menstrual cycles and had been attempting to conceive for six months without contraceptive use. Her obstetric history included one previous spontaneous abortion at six weeks of gestation, managed by surgical evacuation four years earlier. She had no notable medical history, prior abdominopelvic surgeries, or current medications, and reported no substance use.

Two weeks before presenting to our center, the patient sought care at another facility for a missed period. At that time, her β‐hCG level was 103 IU/L, suggestive of a possible pregnancy. Subsequent β‐hCG measurements showed an increase to 224 IU/L one week later and 826 IU/L another week after that. Transvaginal ultrasonography performed at the initial center also reported the presence of a left adnexal mass suspected of an EP mass. The patient was subsequently referred to our center for further evaluation and management.

On initial presentation to our center, the patient denied abdominal pain, vaginal bleeding, or discharge. She was hemodynamically stable, with a blood pressure of 110/70 mmHg, a heart rate of 82 beats per minute, and a temperature of 36.5°C. Physical examination revealed no abdominal tenderness, rebound tenderness, or palpable masses. Pelvic examination was unremarkable, with no cervical motion tenderness, vaginal bleeding, or discharge. Transvaginal ultrasonography showed an empty uterine cavity with an endometrial thickness of 5 mm and a left adnexal mass measuring 15 × 10 mm^2^, located between the left ovary and uterus, highly suggestive of an EP. Laboratory testing revealed a β‐hCG level of 471 IU/L, declining from a previous value, indicating potential spontaneous resolution. Given her stability and decreasing β‐hCG trend, the medical team opted for expectant management with serial β‐hCG monitoring.

However, two days later, the patient presented with acute lower abdominal pain and moderate left‐sided pelvic discomfort, without vaginal bleeding. She was mildly tachycardic, with a heart rate of 93 beats per minute, blood pressure of 115/75 mmHg, and temperature of 37.1°C. Examination revealed tenderness in the left lower quadrant without rebound tenderness, guarding, or palpable masses. Her serum β‐hCG had risen to 1500 IU/L. Repeat ultrasonography showed a 20‐mm gestational sac in the left ampullary region and mild free fluid in the posterior cul‐de‐sac, indicating progression of EP, but no fetal cardiac activity. Concerned about potential tubal rupture, surgical intervention was advised. After comprehensive counseling on the procedure, risks, and alternatives, verbal and written informed consent was obtained, and the patient was transferred to the operating room.

## 3 Methods

**FIGURE 1:**
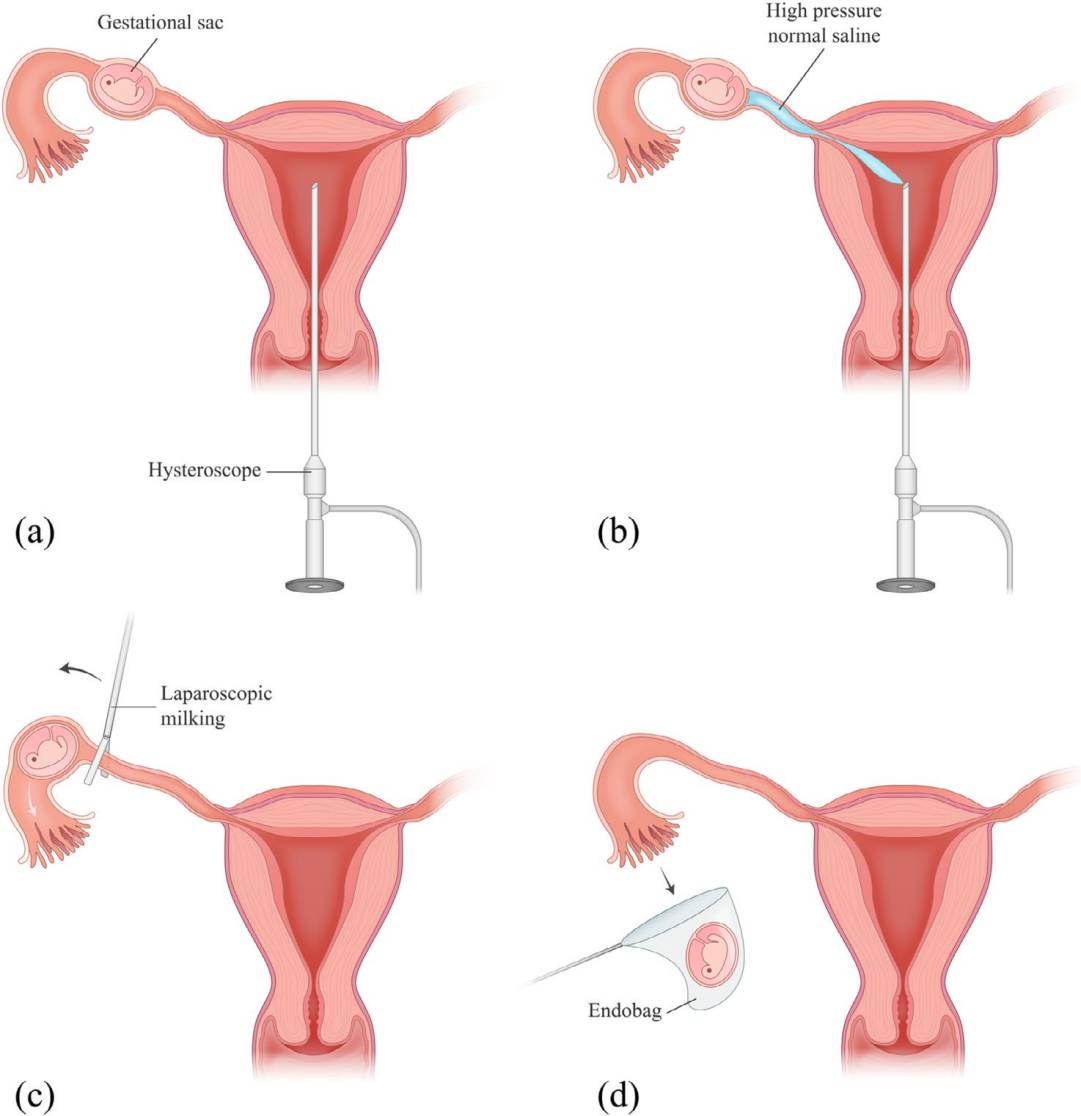
Schematic representation of the surgical procedure employed for the removal of an EP mass located in the ampullary segment of the fallopian tube. (a) Insertion of the hysteroscope into the uterine cavity. (b) Application of hydrostatic pressure using normal saline, delivered via the hysteroscope, to detach the pregnancy mass from its implantation site in the fallopian tube. (c) Laparoscopic milking to guide the detached pregnancy mass toward the fimbrial end of the fallopian tube. (d) Expulsion of the pregnancy mass through the fimbrial end, followed by its retrieval using a laparoscopic endobag.

The patient was positioned in the dorsal lithotomy position. Under general anesthesia, the surgical field was prepared and draped sterilely. A four‐port laparoscopic approach was employed, consisting of a 10‐mm umbilical port for camera placement, two 5‐mm ports in the right and left lower quadrants for instrumentation, and a 10‐mm suprapubic port for the endobag. Initial laparoscopic examination revealed a distended left fallopian tube in the ampullary region, measuring 2–3 cm, with no evidence of active bleeding or significant hemoperitoneum. The right fallopian tube, both ovaries, and the remainder of the pelvis appeared normal. Given the absence of fallopian tube rupture and the patient's strong preference for preserving her fallopian tubes, we employed a novel technique to remove the EP without performing a salpingectomy or making an incision into the fallopian tube. This approach involved laparoscopic‐assisted hysteroscopic tubal flushing to detach and extract the ectopic mass while preserving the integrity of the fallopian tube (Figure 1).

**FIGURE 2:**
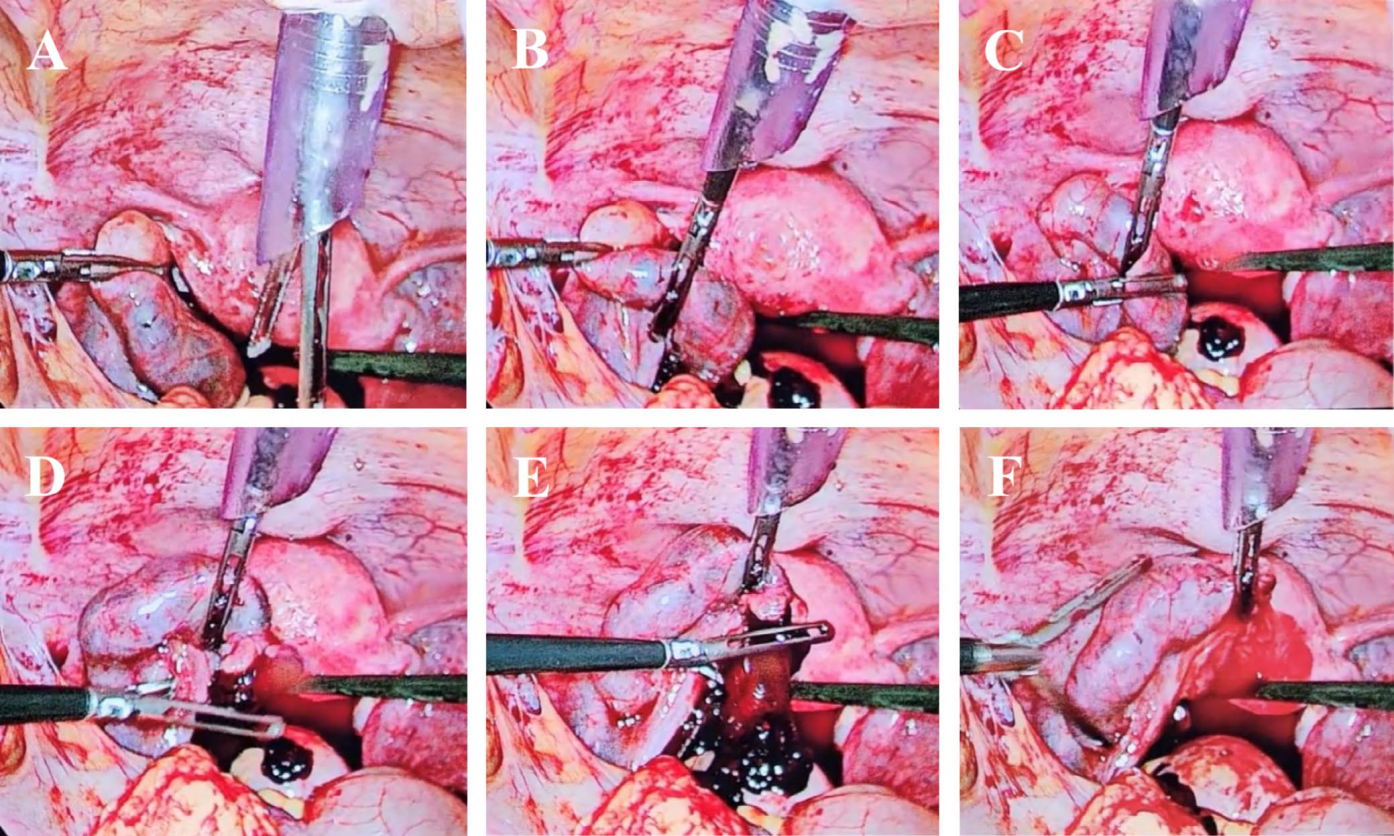
The laparoscopic view of the procedure. (A) High‐pressure normal saline infusion via the hysteroscope detached the pregnancy mass from the ampullary segment of the left fallopian tube. (B, C, D, and E) Laparoscopic milking of the fallopian tube guided the detached pregnancy mass toward the fimbrial end. (F) Following the milking process and release of pressure from the fallopian tube, the remaining pregnancy products, along with stored normal saline from the hysteroscope, were rapidly expelled through the fimbrial end, which were subsequently collected using a laparoscopic endobag.

A hysteroscope was inserted into the uterine cavity, and normal saline (0.9%) was infused to perform the tubal flushing. The infusion pressure was increased from the conventional 100 to 200 mmHg, with fluid flow rates maintained at 500 mL/min. This controlled hydrostatic pressure successfully dislodged the EP products toward the fimbrial end. Laparoscopic tubal milking was then gently performed to facilitate the complete passage of the products through the fimbrial opening, and the products were retrieved using a laparoscopic endobag (Figure 2, Supporting Information). The total operative time was 30 min, with an estimated blood loss of 50 mL. The final inspection confirmed adequate hemostasis, no visible damage to the fallopian tube, no active bleeding, and no requirement for electrocautery to maintain hemostasis. The patient tolerated the procedure well and was transferred to the recovery room in stable condition.

Specimens from the extracted tissue obtained from the left fallopian tube via laparoscopy were sent for histopathological examination. The report described tan tissue measuring 2 × 1 cm^2^, with villus‐like structures and areas of hemorrhage, indicative of products of conception and confirming the diagnosis of a tubal EP.

## 4 Conclusions and Results

Postoperative assessments showed a decline in β‐hCG levels to 1041 and 989 IU/L on postoperative days one and three, respectively. By day six, however, β‐hCG levels rose to 1094 IU/L, suggesting residual ectopic pregnancy tissue. Following protocols, a single intramuscular methotrexate dose (50 mg/m^2^; Trexoma, Nanoalvand, Tehran, Iran) was administered. Subsequent monitoring showed a β‐hCG reduction to 807 IU/L on day four post‐methotrexate and 496 IU/L on day seven, eventually reaching 1.2 IU/L within five weeks.

**FIGURE 3:**
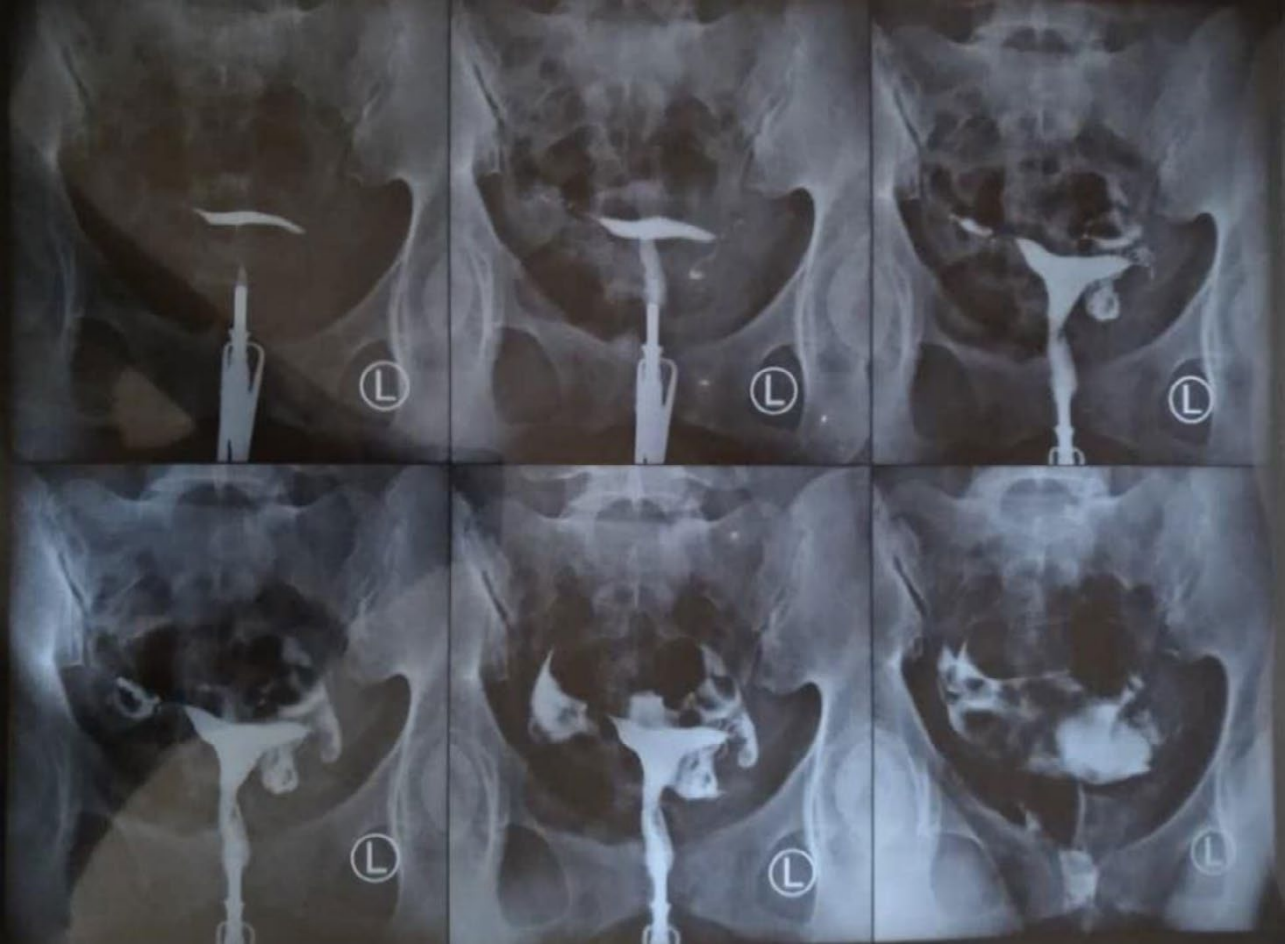
Three‐month follow‐up hysterosalpingography showing a normal uterine cavity and bilateral fallopian tube patency with appropriate caliber and peristalsis.

At three months, the patient reported regular cycles and had an unremarkable physical and pelvic exam. Hysterosalpingography revealed a normal uterine cavity and bilateral tubal patency with appropriate caliber and peristalsis, confirming intact function and structure of the fallopian tubes post‐treatment (Figure 3).

## 5 Discussion

In this case, hysteroscopic application of hydrostatic pressure was successfully used to treat a tubal EP, resulting in complete resolution while preserving fallopian tube integrity. This outcome suggests that laparoscopy‐assisted tubal flushing of EP tissue could potentially be a valuable alternative for managing unruptured tubal EPs in patients requiring surgical intervention but seeking to preserve the structure and function of their fallopian tubes. This novel minimally invasive approach was employed due to the surgical indication for EP management, coupled with the patient's strong preference for tubal preservation.

A review of the literature reveals that efforts to develop effective conservative surgical treatments for EP culminated in the first documented application of hydro‐dissection for pregnancy mass removal in 2007 [13]. This pioneering case involved a patient with a heterotopic triplet pregnancy, consisting of a twin intrauterine gestation and a ruptured tubal EP, rendering methotrexate therapy contraindicated. To address the extrauterine pregnancy, the patient underwent unilateral laparoscopic salpingostomy with hydro‐dissection for EP mass extraction. This approach achieved successful outcomes, enabling the continuation of the viable intrauterine pregnancies [13].

Moreover, a similar tubal flushing technique was previously reported as a tube‐preserving treatment for two ampullary ectopic pregnancies—one bilateral and one unilateral. In this procedure, vasopressin injection proximal to the implantation site expelled conception products through the fimbrial end. Both cases demonstrated successful outcomes with significant β‐hCG reduction at two‐week follow‐up [14].

In another study, Alicyoy et al. utilized laparoscopic salpingostomy in conjunction with hydro‐dissection for the excision of a tubal pregnancy mass [15]. Similarly, another case series reported favorable outcomes in two patients with tubal EPs accompanied by hemoperitoneum; hydro‐dissection was employed for mass detachment, followed by laparoscopic milking and removal via salpingostomy [16].

Furthermore, Nezhat et al. presented a case involving a confirmed cornual pregnancy [17], a condition often managed through unilateral salpingectomy combined with resection of the affected cornual section of the uterus [18]. This traditional approach, however, is associated with significant surgical complications and can adversely impact the patient's future pregnancies [19]. This study utilized hydrostatic pressure via hysteroscopy to separate the EP mass from the cornual region in a method more comparable to the present case than others in the literature. This was followed by milking the mass into the fallopian tube for subsequent removal by salpingectomy. Notably, this technique preserved the cornual section of the uterus, allowing the patient to retain an intact uterine structure, potentially not harming further patients' future assisted reproductive treatments [17].

Our case, however, presented a novel and distinct scenario compared to previous reports of EP hydro‐dissection. It is the first to employ hydrostatic pressure via hysteroscopy to detach a tubal mass from the ampullary region, combined with tubal milking to extract the pregnancy through the fimbrial end without incisions. This method preserved the fallopian tube's structure and function, as confirmed by follow‐up hysterosalpingography. However, postoperative β‐hCG levels did not sufficiently decline; necessitating a methotrexate dose to address potential residual tissue. While hysteroscopic hydrostatic removal shows promise for preserving fallopian tube integrity, careful β‐hCG monitoring remains critical to ensure complete resolution and guide further treatment if needed.

Collectively, these findings suggest that EP removal through hydrostatic pressure via hysteroscopy, followed by laparoscopic milking, may be a promising tube‐preserving option for patients needing surgical intervention for EP. However, caution is essential in the milking process. We believe that detaching the EP mass from the tube using hydrostatic pressure before milking likely reduces the risks of tubal rupture, bleeding, and remnant trophoblastic tissue compared to milking alone. Nonetheless, these risks are inherent to the procedure and demand careful attention [20]. Additionally, this technique requires advanced laparoscopic skills and may not be suitable for all surgeons or centers [21].

It is also important to note that hydro‐dissection has been applied successfully for EP mass removal in various anatomical locations. Dennert et al. described a case where an EP with detectable fetal heart activity on the intra‐abdominal surface of the left diaphragm was effectively removed using laparoscopic hydro‐dissection [22]. This approach allowed for the safe detachment of the pregnancy mass without cauterizing this critical organ, facilitating a secure extraction of the mass. Furthermore, Cárdenas‐Suárez et al. reported a cervical EP that progressed despite medical treatment with methotrexate and was subsequently expelled by applying hydrostatic pressure (flushing) via an injection of 5 mL of 1% lidocaine with a 20G needle into the cervix [23]. These varied applications—through hysteroscopy, laparoscopy, and direct intravaginal injection—suggest that hydro‐dissection could be a versatile surgical option for managing EPs in diverse anatomical sites.

Several important limitations of our findings must be acknowledged. As a case report introducing a novel method, these results cannot be considered definitive for EP management and should be interpreted cautiously. Larger controlled studies are required to establish the safety and efficacy of this approach. We should also highlight that the described procedure itself carries inherent risks that warrant consideration. Both high‐pressure tubal flushing and tubal milking may potentially cause tubal rupture and bleeding [20, 24]. While our stepwise approach—applying hydrostatic pressure to detach the gestational tissue, followed by careful milking toward the fimbria—was designed to minimize risks, these techniques require significant operator expertise. Further research is needed to establish optimal parameters for hydrostatic application, including pressure levels, fluid volume, and directionality to maximize safety.

Our method represents an evolution of previously reported techniques [14] by combining laparoscopic milking with hysteroscopic tubal flushing. However, as demonstrated by our case where postoperative β‐hCG levels unexpectedly increased, requiring methotrexate administration, this approach may not always ensure complete trophoblastic tissue removal. Close postoperative β‐hCG monitoring remains essential, and future studies should investigate technical refinements to improve complete tissue evacuation. In addition, when considering tube‐preserving options, salpingotomy and salpingostomy remain established alternatives with demonstrated efficacy [21]. However, these procedures carry risks of incomplete tissue removal and recurrent EP [10, 11]. Our method may offer advantages for patients strongly opposed to tubal incision, but rigorous comparative studies are needed to properly evaluate its clinical role relative to existing techniques.

Collectively, our case suggests that laparoscopy‐assisted hydrostatic pressure for tubal EP mass removal could potentially serve as a promising alternative to salpingectomy or salpingostomy for patients seeking to preserve their fallopian tubes intact. However, these findings require validation through larger future studies to assess the efficacy, safety, and long‐term outcomes of this technique.

## Author Contributions

**Azadeh Tarafdari:** conceptualization, data curation, investigation, project administration, resources, supervision, validation, writing – review and editing. **Marjan Ghaemi:** resources, writing – review and editing. **Najmeh Nasiri Khormoji:** investigation, writing – original draft. **Amirali Barkhordarioon:** data curation, writing – original draft. **Shiva Hadizadeh:** investigation, writing – original draft. **Mohammadamin Parsaei:** investigation, methodology, resources, software, validation, writing – original draft.

## Ethics Statement

We strictly adhered to the principles of the Declaration of Helsinki throughout the whole study process. After providing a thorough explanation of the necessity of the therapeutic procedure and its potential adverse events, informed verbal and written consent was obtained from the patient prior to the procedure.

## Consent

Written and formal consent for the publication of this case report was obtained from the patient.

## Conflicts of Interest

The authors declare no conflicts of interest.

## Supporting information


**Data S1:** ccr370812‐sup‐0001‐Supinfo.pdf.

**Video S1:** Laparoscopic milking of the EP product from the fimbrial end of the fallopian tube.

## Data Availability

Data sharing is not applicable to this article as no datasets were developed or analyzed during the study.
