# Supplementary figures and images for: Hysteroscopic Tubal Flushing Combined With Laparoscopic Milking for Ectopic Pregnancy Removal: A Case Report and Literature Review
Source: Clin Case Rep. 2025 Aug 19;13(8):e70812. doi: 10.1002/ccr3.70812 (PMC12364717; doi:10.1002/ccr3.70812)

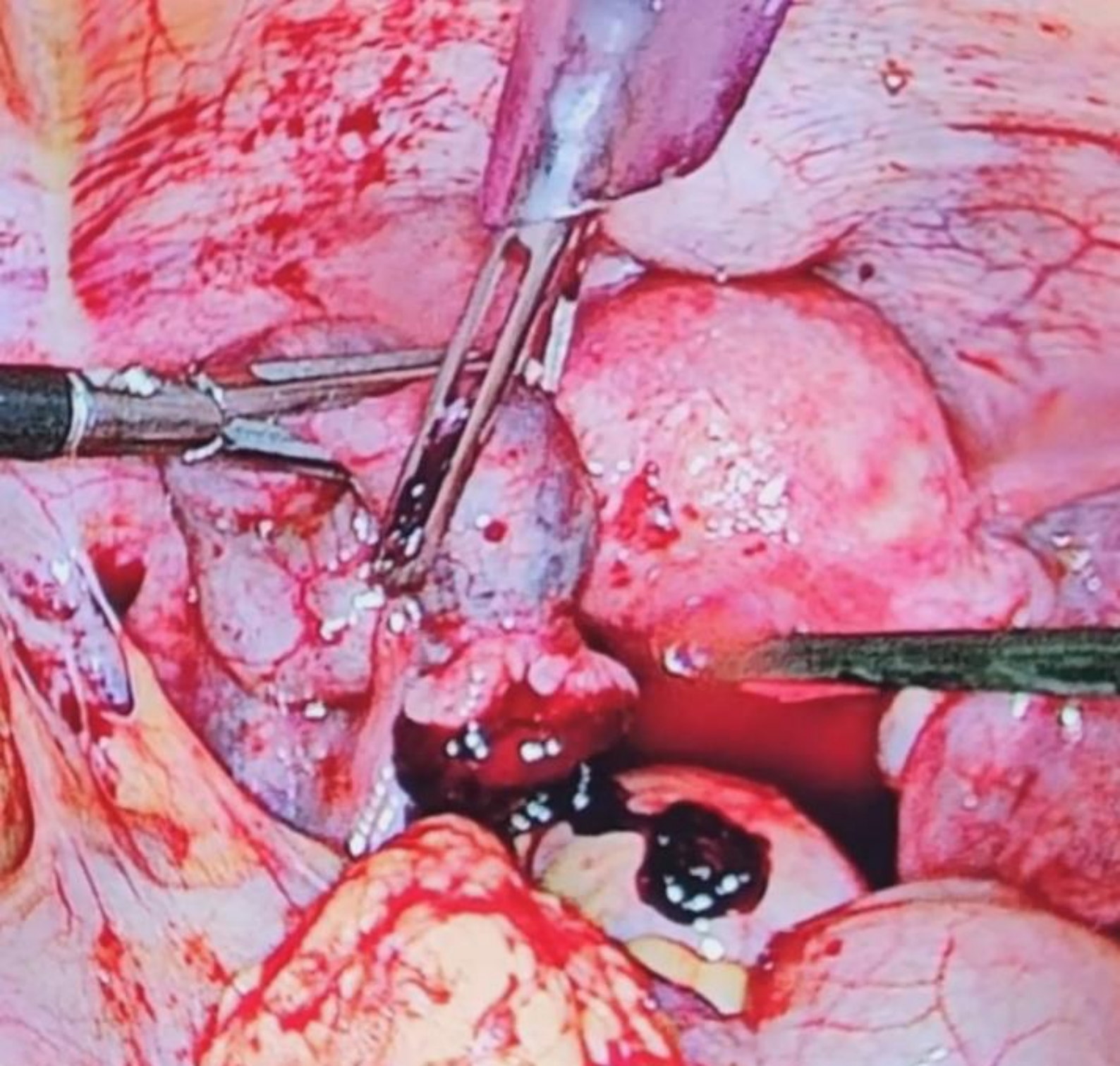

Supplement: Supplementary file 1 — Data S1: ccr370812‐sup‐0001‐Supinfo.pdf. [file CCR3-13-e70812-s002.pdf]
